# Reduced density and visually complex apiaries reduce parasite load and promote honey production and overwintering survival in honey bees

**DOI:** 10.1371/journal.pone.0216286

**Published:** 2019-05-23

**Authors:** Travis L. Dynes, Jennifer A. Berry, Keith S. Delaplane, Berry J. Brosi, Jacobus C. de Roode

**Affiliations:** 1 Department of Environmental Sciences; Emory University, Atlanta, GA, United States of America; 2 Department of Entomology; University of Georgia, Athens, GA, United States of America; 3 Department of Biology; Emory University, Atlanta, GA, United States of America; University of California San Diego, UNITED STATES

## Abstract

Managed honey bee (*Apis mellifera*) colonies are kept at much greater densities than naturally occurring feral or wild colonies, which may have detrimental effects on colony health and survival, disease spread, and drifting behavior (bee movement between natal and non-natal colonies). We assessed the effects of a straightforward apiary management intervention (altering the density and visual appearance of colonies) on colony health. Specifically, we established three “high density / high drift” (“HD”) and three “low density / low drift” (“LD”) apiary configurations, each consisting of eight bee colonies. Hives in the HD apiary configuration were of the same color and placed 1m apart in a single linear array, while hives in the LD apiary configuration were placed 10m apart at different heights, facing outwards in a circle, and made visually distinctive with colors and symbols to reduce accidental drift between colonies. We investigated disease transmission and dynamics between the apiary configurations by clearing all colonies of the parasitic mite *Varroa destructor*, and subsequently inoculating two randomly-chosen colonies per apiary with controlled mite doses. We monitored the colonies for two years and found that the LD apiary configuration had significantly greater honey production and reduced overwinter mortality. Inoculation and apiary management intervention interacted to affect brood mite levels, with the highest levels in the inoculated colonies in the HD configuration. Finally, foragers were more than three times more likely to drift in the HD apiary configurations. Our results suggest that a relatively straightforward management change–placing colonies in low-density visually complex circles rather than high-density visually similar linear arrays–can provide meaningful benefits to the health and productivity of managed honey bee colonies.

## Introduction

There is broad concern over ongoing managed honey bee (*Apis mellifera*) declines. For example, estimates indicate a decline of 61% in the number of managed colonies in the US from 1941 to 2008 due to socioeconomic factors that cause high colony turnover and increased mortality rates [1,2]. Managed colonies are typically kept at a proximity and density that are many orders of magnitude higher than their feral or wild counterparts, which commonly range in density from 1–6 colonies per km^2^ [3,4]. Managed apiaries, in contrast, are densely arranged with colonies typically spaced ≤1m apart [5] and may have 40 colonies in a small space. Such dramatic altering of densities may have serious implications for colony health and survival, disease transmission, and drifting behavior (i.e. when bees enter a non-natal colony).

Population density has been studied as a key factor in ecological relationships going back to Malthus [6], who first described density-dependent mortality and fecundity relationships. Density is known as an important driver of population dynamics across many taxa including insects [7], fish [8], plants [9], and mammals [10]. Density can also be an important modulator of other ecological factors including landscape patterns such as patch size [11] and ecological interactions such as the well-studied effects of prey density on predator consumption rates [12]. Studies have shown in other social insect species that competition for foraging space is indicated in how close ant colonies are distributed [13] and worker ants in crowded colonies expend more energy which may impact colony performance and fitness [14].

In agricultural systems high densities are common, and crowding can have negative consequences on animal performance. For example, high stocking densities can increase the regulation of stress genes and down-regulate immune genes in fish [15], while cows in high-density management settings decrease the amount of time they spend feeding [16]. In honey bees, intracolony crowding can have detrimental effects on colony productivity and bee lifespan [17,18]. Crowded foraging conditions can also initiate signals to stop foraging or decrease the recruitment of new foragers, thus reducing the foraging efficiency of the colony [19,20]. Further, intercolony crowding could have a detrimental effect on homing errors in drone bees and increase parasite loads [5]. In contrast, low-density apiaries could have a negative impact on the frequency in mating for polyandrous honey bee queens [21].

Both theoretical and empirical ecological studies show that population density is also a key factor in driving disease ecology and dynamics [22,23]. Disease ecology predicts that higher host density and greater mixing of host populations will result in greater disease transmission and disease burdens [24] and can lead to the evolution of increased parasite virulence [25]. In the honey bee system it is posited that increased colony density and transmission likely contribute to pathogen and pest virulence evolution [26]. The parasite component of this study focused on the obligate ectoparasitic mite *Varroa destructor* because it is posited to be the greatest biotic threat facing honey bees [27]. *V*. *destructor* parasitism has also been shown to influence honey bee homing ability and could affect drifting behavior and disease spread [28].

In a typical apiary layout, colonies are placed close together, aligned in a row with the entrances facing the same direction, and painted the same color [5]. In this management system there are high densities of honey bee colonies, crowding of foragers, and substantial levels of mixing of bees between these colonies. This mixing can occur intentionally (transferring frames to equalize colony strength), or unintentionally through higher rates of bee drifting into visually similar colonies [5,29]. Given that all of these factors are consistent with negative effects of apiary density and configuration [5], we hypothesized that standard beekeeping management practices will increase competition for floral resources between colonies, result in greater disease burdens and transmission, and negatively affect colony health, productivity, and survival. Specifically, we predicted that *V*. *destructor* levels in apiaries in high density configurations would increase faster and would maintain a higher mite burden throughout the experiment compared to low density configurations. Further, we expected colonies in the high density configurations to have lower colony strength (as measured by adult and brood production as well as honey production) and survival, as well as greater *V*. *destructor* burdens, *V*. *destructor* transmission and worker bee drifting. Finally, we expected to observe higher drifting rates in apiaries with the high density configuration relative to apiaries with the low density configuration.

## Materials and methods

### Overview

**Fig 1:**
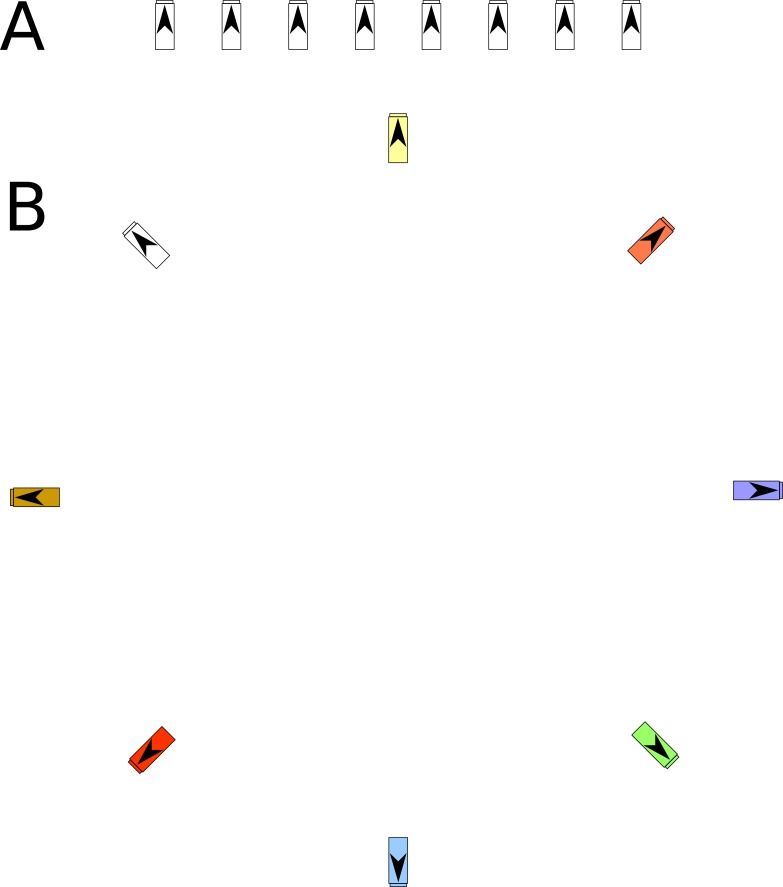
Scale representation of apiary configurations from above. **A** shows a HD configuration and **B** shows a LD configuration. Each configuration was replicated 3 times. The direction of the arrow indicates the colony entrance.

In order to determine the effect of honey bee apiary configuration (a combination of colony spacing, arrangement, and visual complexity) on colony health, parasite burden, and bee drifting, we carried out a two-year study that compared a linear high-density visually similar (high density, hereafter “HD”) colony configuration with a circular low-density visually complex (low density, hereafter “LD”) configuration. We established three HD apiary configurations and three LD apiary configurations in June 2015 around Athens, Georgia, USA, maintained by the University of Georgia Honey Bee Lab. Each apiary was at least 3km away from the nearest apiary and consisted of eight colonies initially housed in standard five-frame Langstroth nucleus hive boxes, for a total of 48 colonies. We arranged HD colonies in a linear array with 1m between colonies and with all entrances facing in the same direction (Fig 1A). HD colonies were all painted white (i.e. no color variability) and placed at a consistent height (200 cm) above the ground. We chose a circular layout for the colonies in the LD apiaries with 10m between colonies and all entrances facing outwards from the center of the circle (Fig 1B). To maximize bees’ ability to visually distinguish between colonies, we painted the LD colonies different colors, painted different symbols at their entrances, and placed the colonies at three different heights above the ground (6, 200, 400 cm, with the spatially closest colonies at different heights). We initially cleared all colonies of *V*. *destructor* and subsequently inoculated two randomly chosen colonies per apiary with 200 adult mites to reflect naturally occurring mite infestations. We monitored colonies for a period of two years.

We then quantified the effect of varying apiary configurations (HD vs. LD), experimentally crossed with *V*. *destructor* infection on four aspects of honey bee health: 1) colony strength (measured by adult bee population, brood population, and honey production); 2) colony survival; 3) *V*. *destructor* reproduction and spread; and 4) worker-bee drifting behavior.

### Colony strength and mite infection

To minimize variation, we started with highly standardized colonies. We established each colony with a mated queen and 1.1 kg (2.5 lb.) of adult bees, shaken into a package. The queens were all from the same queen breeder in southern Georgia, USA and the adult bees were all from common-source backgrounds. The packages were treated for *V*. *destructor* using two separate methods to ensure maximum possible mite clearance. We first used the powder sugar method [30] to encourage bee grooming and mite dislodgement. The packages were then placed in a dark room overnight at 16.6°C (62°F), and sprayed with sugar water one hour prior to the application of 30mL of an oxalic acid solution [31]. Each package was installed three days later in its randomly-assigned apiary. An initial sticky board was left at each colony for 72 hours to ensure colonies were free of *Varroa destructor* mites. To investigate the combined effect of apiary density, colony arrangement, and apiary visual complexity on parasite dynamics, we randomly selected two colonies in each apiary and inoculated these colonies with 200 adult *V*. *destructor* mites. Mites were collected from source colonies outside of the experiment by sifting powdered sugar over the colony and collecting dislodged mites at the bottom of the colony. We used small natural fibered paintbrushes to place mites on damp coffee filters. We kept mites in an incubator set at 35°C (95°F) until all mites were collected. We then transferred all mites evenly to an open brood frame and waited one minute to ensure mites had attached to the wax cells in a colony. We emphasize that inoculated colonies are closer to normally occurring beekeeping conditions (i.e. a *V*. *destructor* mite burden that would be found in a typical apiary) than the near-complete clearance treatment in our experiment since *V*. *destructor* population dynamics are in a state of “permanent exchange” in areas of high colony density [27]. In a large six year survey Traynor et al. [32] found it is not uncommon for beekeeper colonies to have non-zero mite levels in the spring. Under such conditions it is nearly impossible for colonies to maintain non-zero mite levels, as shown by Delaplane and Hood [33] who reported August average mite populations of 111±69 in colonies in which some colonies in the apiary were continuously mite-treated and others not. To maintain our focus on these original colonies (and their queens), we enacted swarm control on colonies likely to swarm by splitting those colonies. We standardized swarm control to ensure small colonies were not jeopardized by the procedure. A total of 38 out of the 48 colonies were split to prevent swarming, all occurring in March 2016. We employed a Fisher’s exact test to determine that there was not a significant difference (*P* = 0.29) in the number of splits between our treatment groups. During the experiment, we did not conduct any control measures against *V*. *destructor*. Colonies were moved into 10-frame hives and supered as demands for growth and storage space dictated. We fed colonies a syrup solution, a common practice in beekeeping management, that we standardized by giving equal volumes across all colonies regardless of need. We continued the experiment from June 2015 through May 2017, at which point only 12 of the original 48 colonies were still surviving.

### Data collection

#### Colony strength assessments

To determine the effect of apiary density and arrangement on colony health we took periodic health measurements throughout the experiment. We followed the colony strength assessment guidelines described in Delaplane et al. [34] to measure the adult bee population, amount of brood, and amount of honey stored for each colony. We performed these colony assessments seven times over the two years of the experiment. We also recorded the date each colony was found to be dead (no living resident bees) and last known date it was alive for survival analyses.

#### Measuring *V*. *destructor* infestation

We measured *V*. *destructor* infestation levels in three different ways. First, we used an alcohol wash method described by Fries et al. [35]. This method involves destructively sampling 300 bees from a colony in alcohol and counting bees and mites (which detach from the bees allowing easier counting) to get a relative mite level on the adult bee population. Alcohol washes were taken approximately monthly during summer and fall and every 3 months during winter and spring. We took nine alcohol wash samples throughout the experiment. Second, we used sticky boards left at a colony for 72 hours [36], a standard method to evaluate *V*. *destructor* levels in a colony by collecting mites that fall and become entrapped on a board placed at the bottom of a colony. We measured mite levels with sticky boards seven times through the first year of the experiment including one immediately following package installation to confirm colonies were *V*. *destructor* free. Sticky boards were taken approximately every other month during the first year and were not continued in the second year of the experiment for logistical reasons. Third, we measured the mite population in brood cells by opening 100 covered brood cells in each colony and counting the number of mites [37]. The mites in brood cells were examined approximately at 4-month intervals. We measured mite levels in brood cells six times throughout the experiment.

#### Drifting behavior

To quantify potential effects of apiary layout on drifting behavior, we tagged individual bees with uniquely numbered tags and used video cameras located above each colony entrance to record bees entering and leaving [38].We performed the tagging and video capture in September 2015. We tagged newly emerged workers, ensuring tagged bees originated from that colony. Tagging was split into three consecutive weeks. Each week we tagged up to 100 bees in all colonies from one HD apiary and one LD apiary. On days 24 and 25 post-tagging, we recorded five to seven hours of video in one-hour segments at each colony. With computer science collaborators, we developed a Matlab video analysis pipeline called GRAPHITE to examine each frame of video and extract frames containing tags [38]. Using the pipeline, we identified tags in the videos and determined: which colony the tagged bee was from; whether the bee was entering or exiting; and whether the bee was carrying a pollen load.

### Statistical analysis

#### Overview

We explored the effect of two main explanatory variables on several metrics of honey bee health, described above in the data collection section. The explanatory variables were: (1) apiary configuration (the composite measure of apiary density, colony arrangement and colony color and symbol); (2) parasite treatment (cleared versus inoculated: whether the colony was inoculated with a standardized *V*. *destructor* infection at the start of the experiment). We conducted three general classes of analyses, based on the following response variables: 1) colony-level mite infection and colony health parameters; 2) colony-level survival; and 3) drifting behavior.

#### Colony strength and mite infection

Longitudinal repeated measures and nested designs, used in our experiment, can result in temporal and within-subject autocorrelation which violates the assumptions of independence for parametric and linear regression methods. Therefore, we used generalized estimation equations (GEE) to account for repeated measures including autocorrelation. We used the ‘geeglm’ function in the ‘geepack’ package v.1.2–1 [39] in R v.3.4.2 [40] to specify and evaluate the GEE models because it allows for longitudinal data with missing observations. We blocked the data by apiary and colony and utilized an autoregressive (AR1) autocorrelation structure. Each initial model was specified using the two explanatory variables and their interaction. In cases where the GEE was unable to converge, a Wilcoxon signed-rank test was applied to each sample date and a Benjamini-Hochberg procedure was completed to adjust the false discovery rate of testing multiple comparisons.

#### Survival analysis

We performed survival analysis on the colonies using both explanatory variables and their interaction, apiary configuration and colony inoculation status. We also completed a separate winter survival analysis since *V*. *destructor* infection is implicated in reduced winter survival [41,42]. Colonies were inspected periodically throughout the experiment and exact timing of colony death could not be determined. Therefore, we used an interval of date of observed colony death and date of last known colony viability. Given this data structure, we analyzed survival with Cox proportional hazard models with interval censoring via the ‘frailtypack’ package [43] in R, which allows for specification of the equivalent of random effects (apiary only, as single-colony survival over time is the response variable). Since the winter survival data consisted solely of binomial data at a single time point (i.e., survived vs. did not survive through the winter), we employed a separate binomial-errors generalized linear mixed model (GLMM) using the lme4 package [44] in R with random effects for colony identity, nested within apiary. We tested the model for over-dispersion.

#### Drifting data analysis

In order to understand how an inoculated colony status and apiary configuration affect bee drifting behavior, we ran GLMMs using the lme4 package, with counts of drifting events modeled with Poisson errors. The model was tested for over-dispersion. Drifting may also be correlated with proximally located neighbors. We used Mantel correlograms to assess the correlation between drift and relative colony position (nearest neighbor, second nearest neighbor, third nearest neighbor, etc.) with the ‘vegan’ package in R [45], using the progressive correction for multiple comparisons.

## Results

### Overview

We collected extensive data on the strength of the colonies, mite levels, and the movement of individual bees throughout the experiment. The colony health assessments resulted in 202 measurements each of: the adult bee population, brood coverage, and honey storage. In order to evaluate *V*. *destructor* levels throughout the experiment, we collected 316 sticky boards, 279 alcohol washes (each containing approximately 300 worker bees), and 208 counts of mites in the brood (each including 100 brood cells). We recorded and used the GRAPHITE pipeline to process approximately 1200 hours of video tracking individually tagged bees. We observed 120 uniquely tagged individual bees at 242 separate times.

### Colony strength

**Fig 2:**
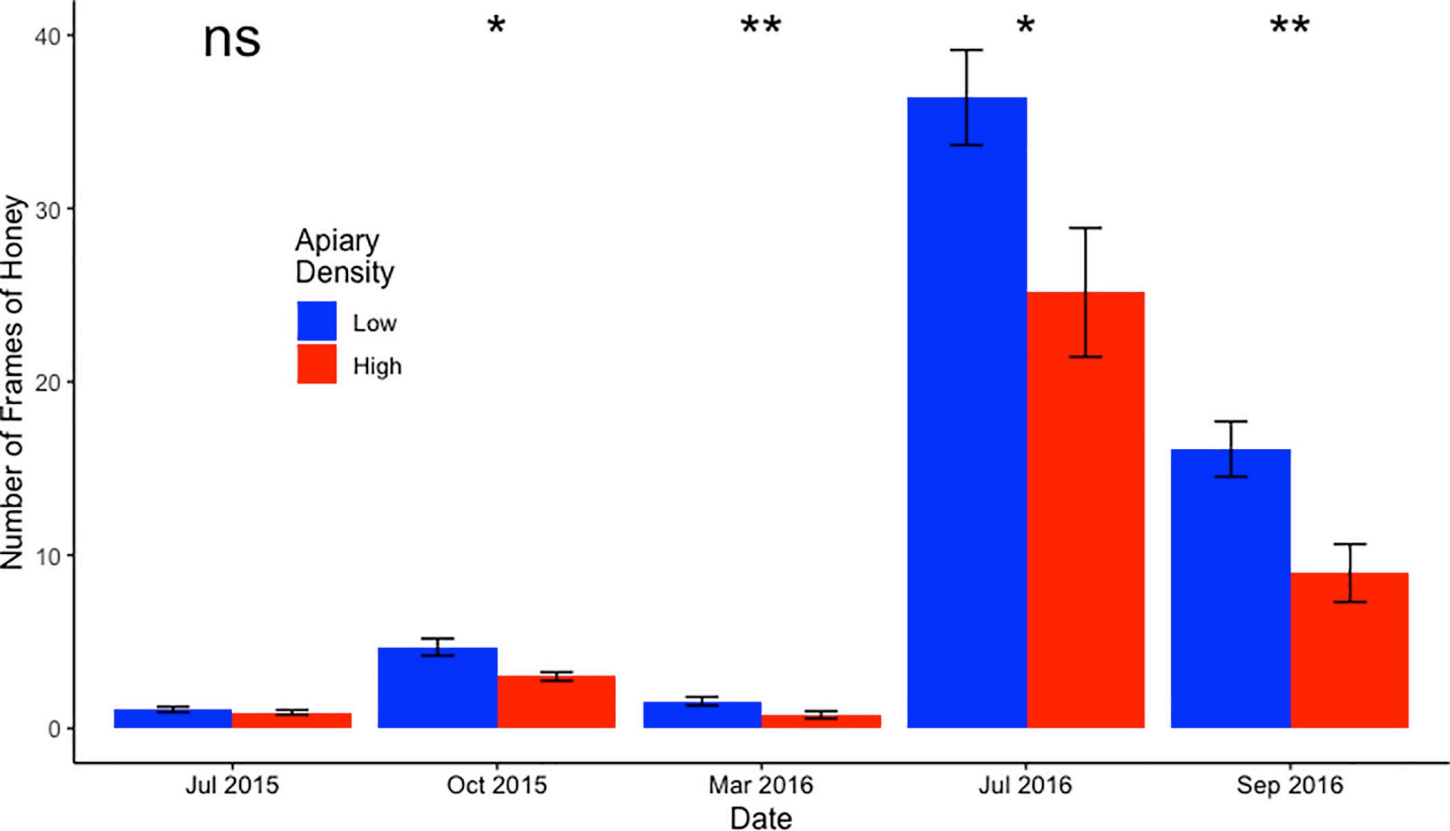
Honey production over time grouped by apiary configuration. A Wilcoxon test was applied to the configuration treatment comparison in each month. The Benjamini-Hochberg procedure was completed to confirm the significance of the multiple comparisons. Error bars represent standard error of the mean. *: P<0.05; **: P<0.01; ns: not significant.

The GEE model of honey storage did not converge, likely due to the very strong seasonal pattern in honey production. A Wilcoxon signed-rank test was completed for each of the sample dates for honey stores and after the Benjamini-Hochberg procedure for multiple comparisons was applied, LD configurations had significantly more honey stores on four of five sample dates (*P* = 0.020, 0.010, 0.040; Fig 2 and S1A Fig), with the only non-significant sample date being the first sample. Neither of the GEE models for the adult bee population and amount of brood (which did successfully converge) had any statistically significant fixed effects (S1B and S1C Fig).

### Mite levels

**Fig 3:**
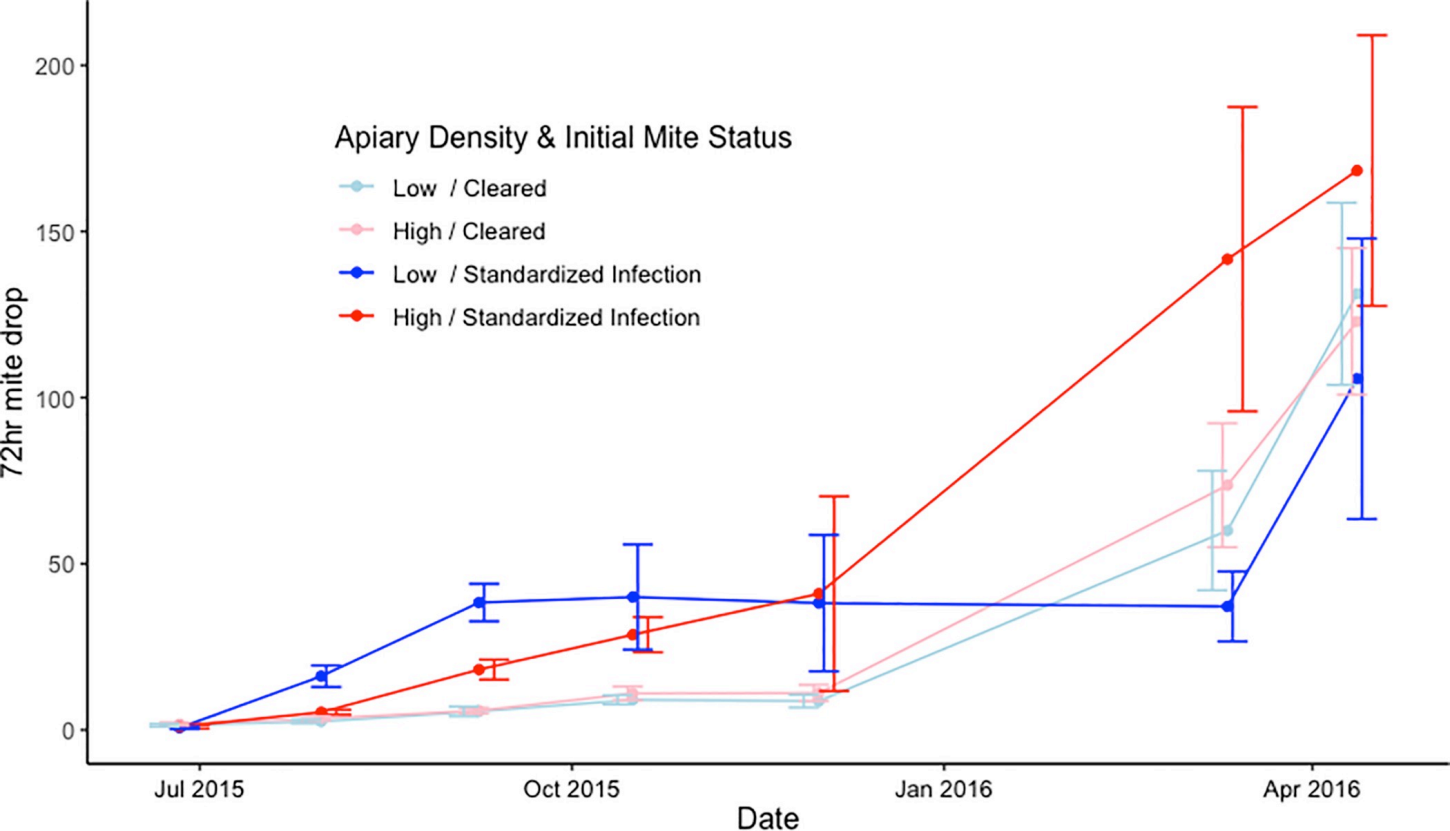
Mite count by sticky board separated by treatment and inoculation over time. Note the cleared colonies in both density treatments stayed at low levels throughout the first winter while inoculated colonies had a steady increase. By the end of the first year however, the cleared colonies had reached the same infection levels as the inoculated colonies. Error bars represent the standard error of the mean. A GEE found a significant positive effect for inoculation on mite numbers (P = 0.0188).

**Fig 4:**
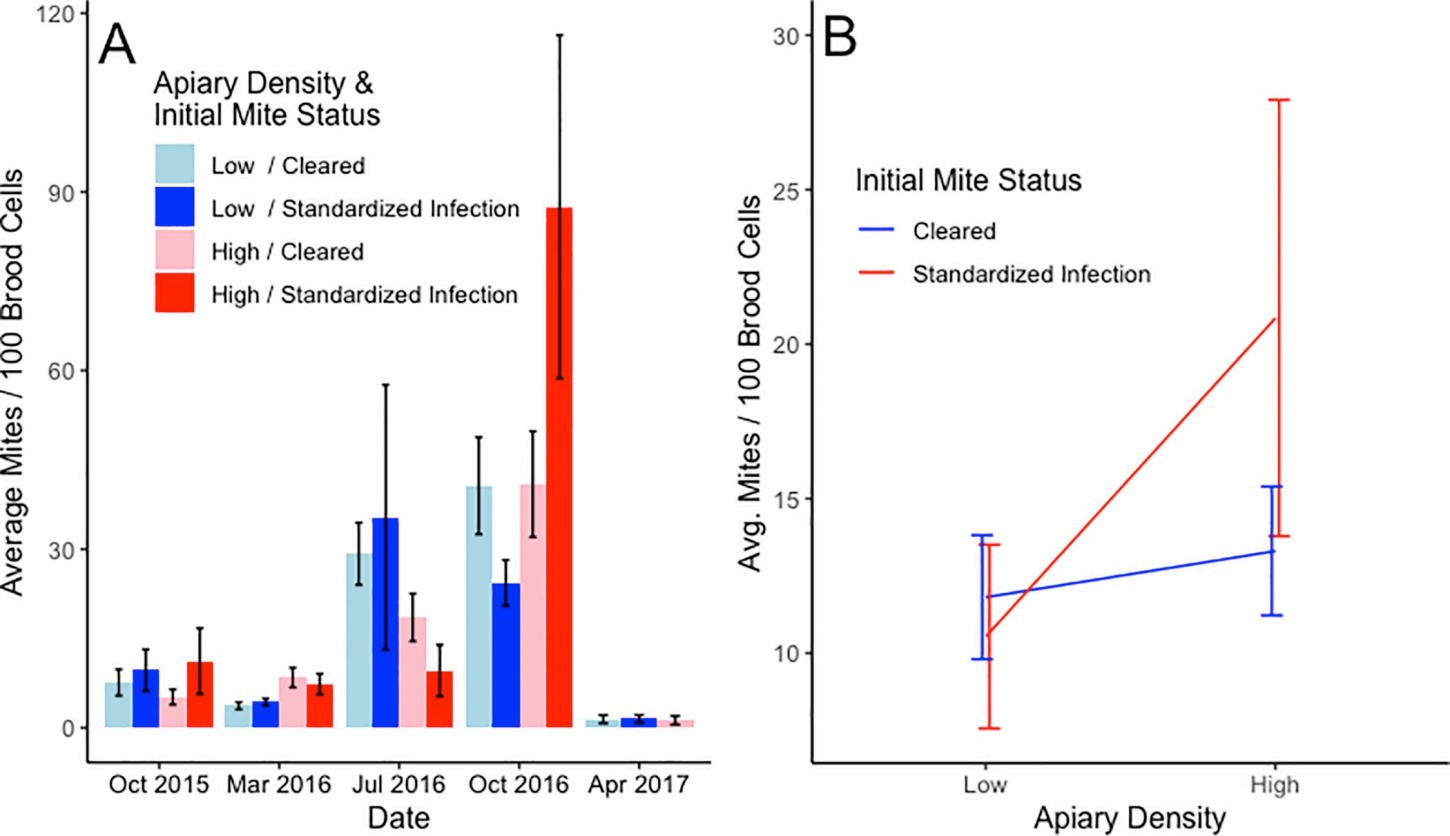
Mites levels in brood. **A.** Mites in 100 brood cells by inoculation status and apiary configuration. **B.** A GEE found a significant interaction (P = 0.0176) between inoculation status and configuration. Error bars in both plots represent the standard error of the mean.

A GEE model of the sticky board data showed a significantly (*P* = 0.0188) positive effect of inoculation on mite levels, but no effect of apiary configuration (Fig 3 and S1D Fig). The GEE model did not show a significant relationship between apiary density or inoculation on mites per 100 brood cells. However, there was a significant interaction (*P* = 0.0176) between inoculation status and apiary configuration. The interaction indicates that that there were significantly more mites in brood when colonies were both inoculated and located in a HD configuration (Fig 4 and S1E Fig). The GEE for the mite levels as assessed by alcohol washes did not have any significant terms (S1F Fig).

### Survival analyses

**Fig 5:**
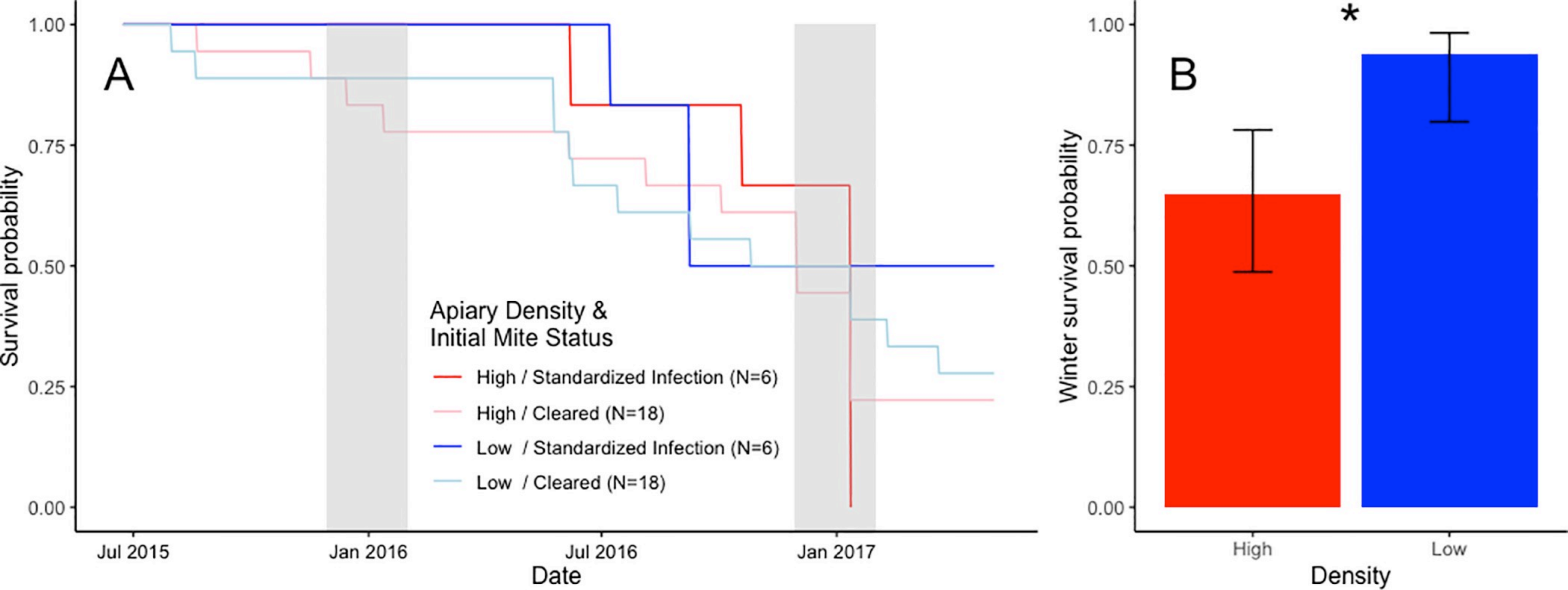
Survival curves and winter survival. **A.** Survival curves by apiary configuration and inoculation status. Gray bars show the winter months of December and January. Note the large drop in survival over the second winter in the HD configurations. **B.** Effect of density on winter survival (Note: a colony surviving until the start of the second winter is counted twice in this figure). A logistic model of winter survival did find that colonies in low density configurations were significantly more likely to survive the winter (P = 0.037). Error bars in B show binomial confidence intervals.

The Cox survival analyses did not show any significant relationship between apiary configuration and inoculation status or their interaction (Fig 5A). However, the binomial-errors GLMM showed that apiary configuration was significantly related to winter survival (*P* = 0.037), with colonies in the lower-density configuration more likely to survive winter (Fig 5B).

### Drifting analyses

**Fig 6:**
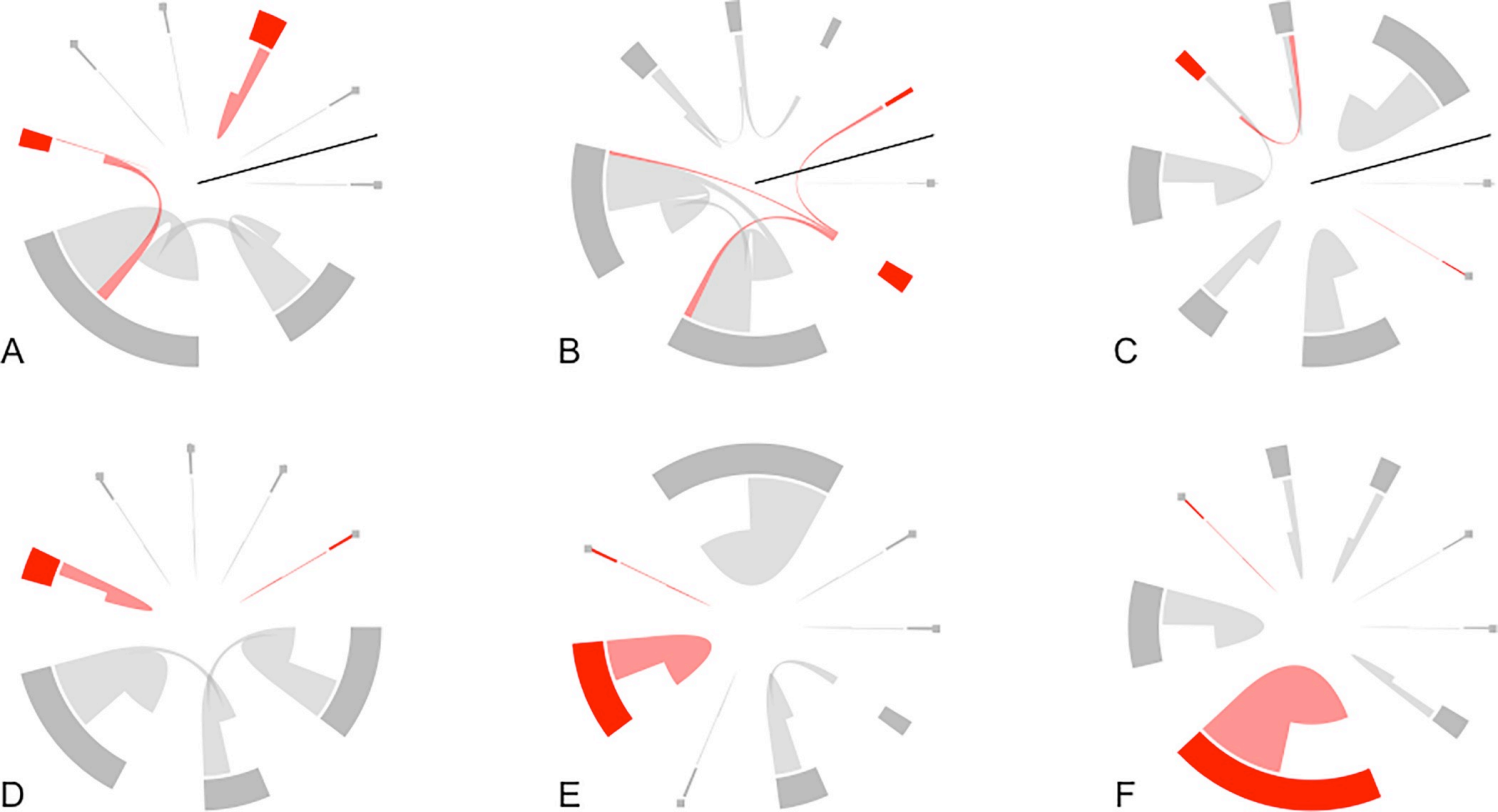
Representation of drift within each of the apiaries. Each panel shows eight colonies as subsections of the circle’s circumference (the width of these subsections indicates number of unique bees observed at colony) and bee movements (individual bees are indicated with thin lines, so that large numbers of observed movement result in large “wedge-like” shapes). Direction of drift is indicated by how close an individual bee movement line is to the colony representation on the circle’s circumference: a bee drifts to a colony where the line is close to the colony and came from a colony where there is a large gap between the line and the colony. Red indicates inoculated colonies while gray indicates cleared colonies. Panels A-C represent the HD configurations; these are represented in a circular format here for comparison to the LD configurations; the black lines in A-C represent where linear arrangement of them would be split to be put back in a straight line. Panels D-F represent drift in the LD configurations. All drift in D-F is to the nearest neighbor. In contrast, there is increased drift in A-C and there are two instances of drift beyond the nearest neighbor (both in B). The inoculation status of a colony was not found to have a significant effect on drift.

Bees in HD configurations (*N* = 134 bees tracked) were significantly (*P* = 0.048) more likely to drift based on a mixed effects model (Fig 6). 25.0% of all tagged bees in the HD configuration drifted, while only 7.5% in the LD configuration (*N* = 89 bees tracked) drifted. On four occasions we observed a drifting bee entering the same (non-natal) colony multiple times, and once we detected a bee that had drifted returning to its natal colony. Nearly all drifting in the HD configuration was to the nearest neighbor (1m) to the focal bee’s natal colony (24 of 26 drifting events), with one drifting event each to the second and sixth-nearest neighbor. In the LD configurations, all three observations of drift occurred at the nearest-neighbor colonies (10m distant). Drifting bees were significantly likely (*P* = 0.047, Mantel correlogram) to drift into the colony nearest their natal colony, drifting to colonies further away was not significant. Mite inoculation status was not significantly related to drifting behavior.

## Discussion

### Overview

Managed honey bee colonies are kept at densities that far exceed naturally occurring densities in feral colonies. Current beekeeping practices which favor these high densities for logistical reasons are predicted to have detrimental effects on disease spread and colony survival [26]. Our present work generated four main findings related to how apiary configuration affected honey bee health. First, the HD configuration significantly decreased honey production. Second, the interaction of inoculation and HD configuration resulted in significantly higher levels of *V*. *destructor* infestation of honey bee brood. Third, HD configurations had a significant detrimental effect on a colony’s winter survival. Finally, drift was significantly increased in HD configurations.

### Colony strength

We found that honey production was decreased in high density configurations (Fig 2). Colony honey hoarding is in general positively associated with adult bee population [46], but as we found no concomitant reductions in either adult bee or brood populations, our present findings are better interpreted as direct effects of either 1) apiary density configuration or 2) increasing numbers of *V*. *destructor* mites.

First, honey bee colonies may operate less efficiently in a HD configuration because of confusion or mixed signaling, leading to lower forager efficiency. For example, one study found that forager crowding prompts foragers to signal to other bees to stop foraging [20].

Second, increasing mite infestations may negatively impact colony honey stores. However, Murilhas [47] could not detect progressively negative effects of mites on brood, adult populations, or honey stores until mite populations reached extreme highs. Moreover, he could detect no direct effects of colony mite level on honey stored per bee per day and concluded that putative effects of mites on colony honey stores are an indirect effect of crashing adult bee populations. Our data indicated increased mite burdens in the brood of inoculated colonies in HD configurations but, as in Murilhas (2002), this occurred late in the experiment. However, in contrast to Murilhas (2002), honey stores in our inoculated, HD configurations were consistently lower throughout most of the experiment. In short, the effects of *V*. *destructor* on honey hoarding, at either the individual or colony level, remain ambiguous. However, it is well-known that *V*. *destructor* mites, in addition to causing pathology themselves, transmit pathogenic viruses [41,48], and it is possible that the observed reduction in honey stores was partly driven by these viruses. Importantly, viruses may have an effect on colony performance by affecting queen behavior and fecundity [49]. Further experiments would be necessary to explore this possibility.

### Mite levels

From initial apiary inoculations of 400 mites (2 colonies x 200 mites), we predicted that *V*. *destructor* levels in both inoculated and uninoculated colonies in apiaries in high density/visually simple configurations would increase faster and remain higher throughout the experiment compared to low density/visually complex configurations. This did not happen, in spite of previous field work supporting this expectation. For example, one study found increased invasion rates and population levels of *V*. *destructor* in colonies that were surrounded by higher densities of colonies [50]. Another showed significantly fewer mites in apiaries in which colonies were spaced 100m apart compared to apiaries with inter-colony distances of 0m or 10m [51]. And genetic analyses are consistent with substantial horizontal transmission of mites between colonies under conditions of intense beekeeping [52].

We did, however, detect a significant interaction between inoculation status and apiary configuration in brood mite levels (Fig 4B) such that highest mite levels occurred in inoculated colonies in HD configurations. This result may be driven by the time delay needed for mites to increase to high levels, even if colonies in the higher density apiaries had higher levels of mite transmission relative to low density apiaries.

In our study, inoculating an incipient mite population, whether under HD or LD conditions, caused an elevation of colony mite levels that persisted at least five months, mirroring in reverse the results of Delaplane and Hood [33] who showed season-long benefit from an early (February) treatment of colonies coming out of winter. However, only in HD apiaries did inoculation also result in a larger fraction of mites in brood. As it is only mites in brood that reproduce, the percentage of mites in brood is a positive indicator of a mite population’s fecundity [53]. It is not apparent how apiary density could influence the relative distribution of mites between adults and brood, especially in our case in which brood area was not different across effects. It is interesting to compare these results to those of Nolan and Delaplane [51] who, using a different method to modify apiary configuration, showed higher end of season mite populations in high-density apiaries following incipient apiary inoculations of 300 mites–but failed to show differences in mites in brood. Thus, while landscape-scale regulators appear to be relevant in *V*. *destructor* fecundity and population size, future work is needed to piece together the mechanisms.

### Survival analysis

Interestingly, survival analysis over the course of the entire experiment did not indicate greater mortality for inoculated than cleared colonies or differences between HD and LD configurations. This may have been partly due to other factors. For example, background mortality as driven by failing queens or other factors may have masked infection and density effects on mortality during the early stages of the experiment. In addition, as described above, mite dynamics suggested between-colony transmission of mites, thereby making cleared and inoculated colonies more equal in mite numbers over time.

However, when survival analyses were restricted to overwintering mortality, we did find a significant effect of apiary configuration. Indeed, while exactly 50 percent of inoculated colonies survived through two winters in LD apiaries, not a single inoculated colony survived through two winters in the HD configurations (Fig 5A). Our determination that winter survival was significantly greater in lower density apiaries is an important finding for beekeepers. Winter mortality is currently one of the greatest challenges facing beekeepers in moderate to cold temperate regions of the globe [42,54,55], and our experiment suggests that this mortality can be significantly reduced by managing colonies at LD configurations. There are at least two factors that could have contributed to this increased survival in our experiment. First, LD configurations had higher honey production. Since colonies in temperate regions need adequate honey supplies to survive the winter [56] it follows that the increased honey production could play a role in increased survival. While the colonies in our study were fed supplemental sugar syrup (standardized across colonies), as is standard practice in beekeeping management, there could have been population-based variation among colonies in their ability to convert this to honey stores [46], along with variation in flower foraging rates, as well as differences in consumption rates which we did not measure. Second, the significantly higher mite levels in capped brood for inoculated colonies in HD configurations in the sample date before the second winter (Fig 4A) may have contributed to the 100 percent mortality of the remaining colonies that winter.

Our results are consistent with another study, in which overwinter survival was also found to be significantly lower in a crowded apiary compared to a group of dispersed colonies [5]. However, in that study, bee swarming was not suppressed, making that work potentially less applicable to common beekeeping practices.

### Drifting

It has long been known that distance, entrance direction, and apiary layout can affect drifting behavior in honey bees [57]. We used these insights to minimize drifting in our LD configurations by placing colonies 10m apart in a circle, at different heights and with colonies facing outwards and being painted different colors and marked with different symbols. These measures were effective, reducing drift from 25% in the HD to 7.5% in the LD apiaries. This quantification is important for managers considering how to slow down disease transmission and for disease modelers working to parameterize the effect of space on disease spread. These observed drifting rates fall within the large ranges in the proportion of drifting individuals (0–89%) found in other studies [29,58]. Our observation of four bees staying in non-natal colonies after drifting suggested that drifting bees could permanently switch colonies after drifting rather than go back and forth, though these are very small sample sizes. Our experiment also showed that the majority of drifting occurred between nearest neighbors. Together these findings suggest relatively lower amounts of disease transmission, compared to the alternatives of bees drifting back and forth between natal and non-natal colonies, or bees randomly drifting to any colony which causes mites to be spread greater distances from the original inoculation. Although our experiment was not able to directly quantify between-colony transmission of mites through drift, it is highly likely that increased rates of drift increase disease transmission. The video-tracking of drifting bees we used here [38] provides a fruitful method to further study the role of drifting in disease transmission.

### Conclusion

Current apiary management utilizes colonies in configurations similar to our HD treatment (closely spaced, visually similar colonies in linear arrays at the same height) for practical and logistical reasons. However, our experiment, using replicated HD and LD configurations, shows that typical management practices (our HD configuration) can be detrimental to colony-level health and productivity. Our results suggest that by lowering the apiary density and making colonies visually distinctive, beekeepers can increase colony productivity, reduce overwinter mortality, and potentially reduce the spread of diseases within the apiary through reduced drift. These steps are relatively modest and should be possible to implement in many beekeeping operations.

## Supporting information

S1 Fig *A-F*. **Supplementary figures for “Reduced density and visually complex apiaries reduce parasite load and promote honey production and overwintering survival in honey bees”, Dynes et al.**
(DOCX)
