# Supplementary material for: Reduced density and visually complex apiaries reduce parasite load and promote honey production and overwintering survival in honey bees
Source: PLoS One. 2019 May 23;14(5):e0216286. doi: 10.1371/journal.pone.0216286 (PMC6532956; doi:10.1371/journal.pone.0216286)
Supplement: S1 Fig — A-F. Supplementary figures for “Reduced density and visually complex apiaries reduce parasite load and promote honey production and overwintering survival in honey bees”, Dynes et al. (DOCX) [file pone.0216286.s001.docx]

*S1 Figs A-F.* **Supplementary figures for “Reduced density and visually complex apiaries reduce parasite load and promote honey production and overwintering survival in honey bees”, Dynes et al.**


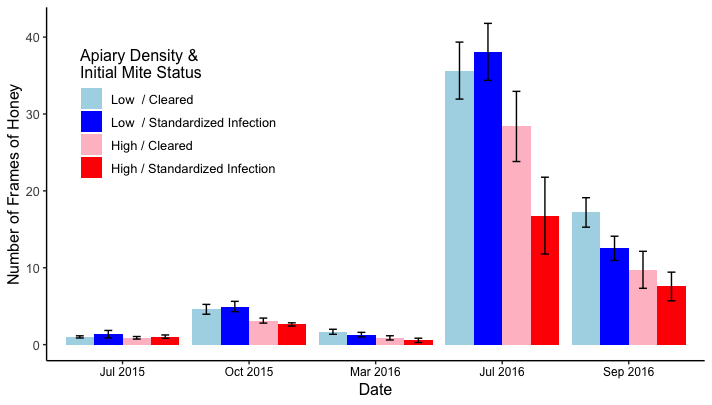


S1 Fig A **Honey Stores.** Average number of frames of honey at each data collection time point by apiary density and initial mite status. Error bars represent standard error of the mean.


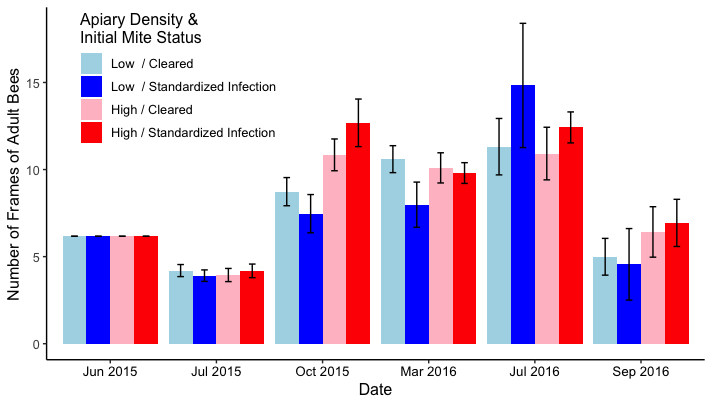


S1 Fig B **Frames of Adult Bees.** Average number of frames of adult bees at each data collection time point by apiary density and initial mite status. Error bars represent standard error of the mean.


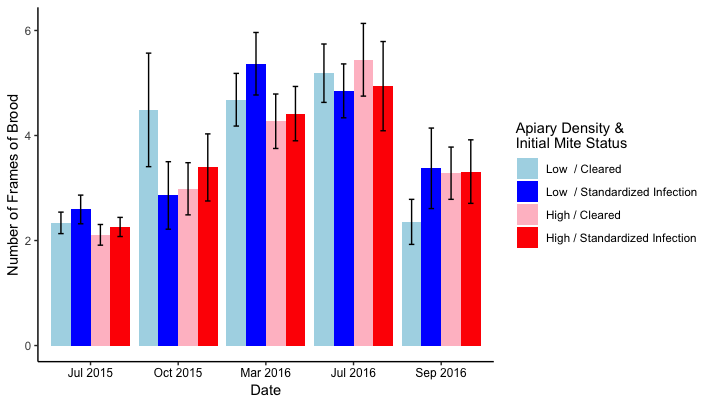


S1 Fig C **Brood Coverage.** Average number of frames of brood at each data collection time point by apiary density and initial mite status. Error bars represent standard error of the mean.


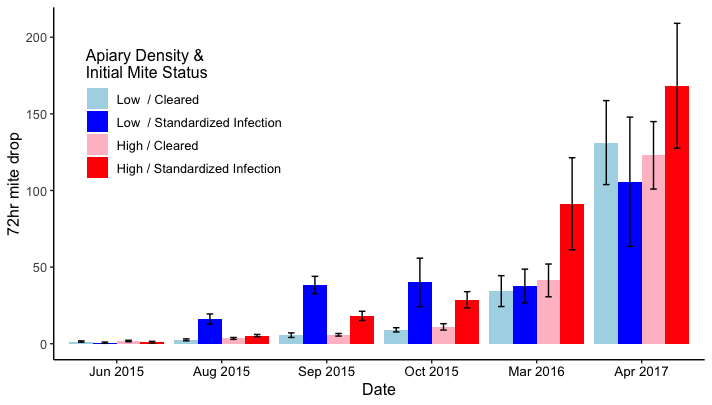


S1 Fig D **Mite levels by Sticky Board**. Average mite count from 72-hour mite drop at each data collection time point by apiary density and initial mite status. Error bars represent standard error of the mean.


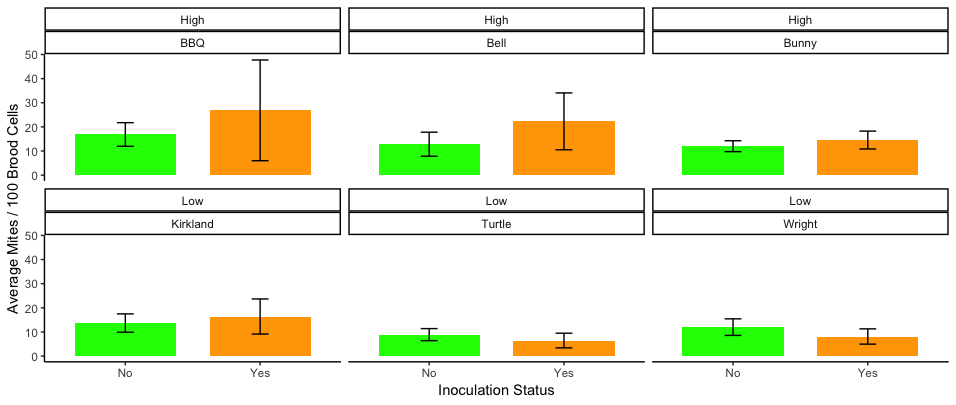


S1 Fig E **Mite Levels in Brood.** Mites in 100 brood cells by density/configuration (High, Low), apiary (named BBQ, Bell, Bunny, Kirkland, Turtle, Wright) and initial mite status (No, Yes). This shows the data in Figure 4b broken down by apiary. Note the higher mite levels in the inoculated colonies located in the high density apiaries (top three figures). Combining the data results in a significant interaction between inoculation status and apiary density (Figure 4b).


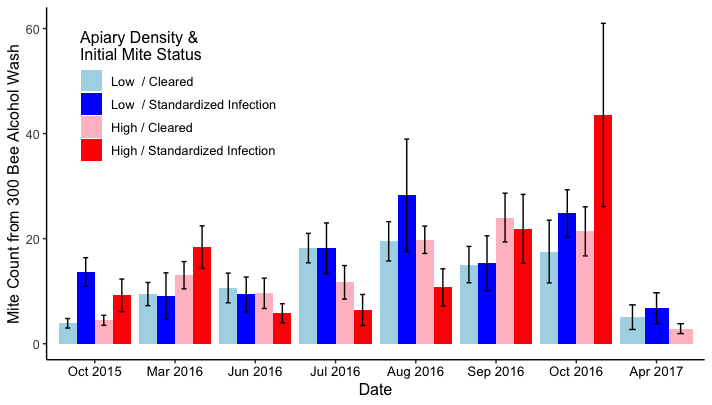


S1 Fig F **Mite levels by Alcohol Wash.** Average mite count from 300 adult bees at each data collection time point by apiary density and initial mite status. Error bars represent standard error of the mean.
